# Whole-genome sequencing and SNV genotyping of ‘Nebbiolo’ (*Vitis vinifera* L.) clones

**DOI:** 10.1038/s41598-017-17405-y

**Published:** 2017-12-11

**Authors:** Giorgio Gambino, Alessandra Dal Molin, Paolo Boccacci, Andrea Minio, Walter Chitarra, Carla Giuseppina Avanzato, Paola Tononi, Irene Perrone, Stefano Raimondi, Anna Schneider, Mario Pezzotti, Franco Mannini, Ivana Gribaudo, Massimo Delledonne

**Affiliations:** 1 Institute for Sustainable Plant Protection, National Research Council (IPSP-CNR), Torino, Italy; 2 Department of Biotechnology, University of Verona, Verona, Italy; 3 Institute for Sustainable Plant Protection, National Research Council (IPSP-CNR), Grugliasco (TO), Italy

## Abstract

‘Nebbiolo’ (*Vitis vinifera*) is among the most ancient and prestigious wine grape varieties characterised by a wide genetic variability exhibited by a high number of clones (vegetatively propagated lines of selected mother plants). However, limited information is available for this cultivar at the molecular and genomic levels. The whole-genomes of three ‘Nebbiolo’ clones (CVT 71, CVT 185 and CVT 423) were re-sequenced and a *de novo* transcriptome assembly was produced. Important remarks about the genetic peculiarities of ‘Nebbiolo’ and its intra-varietal variability useful for clonal identification were reported. In particular, several varietal transcripts identified for the first time in ‘Nebbiolo’ were disease resistance genes and single-nucleotide variants (SNVs) identified in ‘Nebbiolo’, but not in other cultivars, were associated with genes involved in the stress response. Ten newly discovered SNVs were successfully employed to identify some periclinal chimeras and to classify 98 ‘Nebbiolo’ clones in seven main genotypes, which resulted to be linked to the geographical origin of accessions. In addition, for the first time it was possible to discriminate some ‘Nebbiolo’ clones from the others.

## Introduction

The availability of a reference genome in grapevine^1^ has provided in recent years an enormous boost to genetic and functional studies in this species^2,3^. Nevertheless, the use of next-generation sequencing data with the available grapevine reference genome PN40024 may lead to the loss of important information on the interesting characteristics of cultivars. This is due not only to the possible lack of information in the reference genome^4^ because of its highly homozygous nature, but also to the impossibility of the reference genome to represent the entire genetic variability of the species pangenome^5,6^. The *de novo* assembly of plant genomes from short-read sequencing data is challenging^7^ for highly complex polyploid genomes and can lead to highly fragmented genome drafts with no possibility for correctly phasing the haplotypes^8^. However, several projects involving the sequencing or re-sequencing of grapevine cultivars have recently been performed^5,8–14^. There is increasing evidence that genomic variants such as single-nucleotide variants (SNVs), small insertions and deletions (INDELs), inter- and intra-chromosomal translocations and inversions and private genes can contribute to intra-specific variability or to a dispensable genome^15^, unearthing phenotypic characters specific to each cultivar^5,6^.

Throughout history, thousands of grape cultivars have been generated. These cultivars resulted from several processes: domestication from local wild *Vitis vinifera* subsp. *sylvestris* vines, which likely occurred at multiple geographical centres^16^; crosses between domesticated (maybe introduced from other areas) and local wild plants; the historic practice of growing seedlings from spontaneous crosses; and conventional breeding. The selected individuals were then multiplied by vegetative propagation (cutting, layering, or grafting), during which somatic modifications spontaneously occurred and were maintained, thus giving rise to the intra-varietal variability associated with phenotypic variation among grapevine clones^17^. In some cases somatic mutations affecting only some cells led to chimeras, for example periclinal chimeras resulting from mutation of only one cell layer (L1 or L2) of apical meristem. Over the years, the berry colour is a phenotypic character intensively investigated to deepen these mutations, and interesting examples derived from the ‘Pinot’ family and the mutations associated to ‘Pinot noir’, ‘Pinot gris’ and ‘Pinot blanc’^18,19^. Among the thousands of cultivars throughout the world that constitute the germplasm of *V*. *vinifera*, ‘Nebbiolo’ (major synonyms ‘Chiavennasca’ and ‘Spanna’) is among the most ancient and most prestigious wine grape varieties and is renowned for its use in producing high-quality red wines^20,21^. Although cultivated today in different regions of the world (such as California and Australia), the typical cultivation area of ‘Nebbiolo’ is limited to the hilly and mountainous areas of north-western Italy (Piedmont, Aosta Valley, and Lombardy), where outstanding-quality wines such as Barolo, Barbaresco, Gattinara, and Valtellina Sforzato are produced. The first historical quotation of ‘Nebbiolo’ was in 1266 and refers to the castle of Rivoli (Turin surroundings, Piedmont). Between the thirteenth and fourteenth centuries, ‘Nebbiolo’ was mentioned in many other documents related to Piedmont^20,22^, and in the sixteenth century in Valtellina (Lombardy)^23^. Although the parentage of ‘Nebbiolo’ is unknown, great numbers of grape cultivars from these areas are closely related to ‘Nebbiolo’, suggesting its cradle is in north-western Italy or at least has had a long-lasting stay in that region^24^. ‘Nebbiolo’ shows great intra-varietal phenotypic polymorphism resulting in clones (each obtained by vegetative propagation of a single mother plant) with different morphological and physiological characters, such as leaf shape and size, shoot vigour and yield, soluble solids, and the phenolic content of juice at harvest^24^. This may be the reason for the traditional classification of ‘Nebbiolo’ into the so-called subvarieties or biotypes, such as “Lampia”, “Bolla”, “Michet” and “Picoutener”. The large intra-varietal variability is highlighted by the 44 clonal selections officially registered in the Italian National Register of Grape Varieties (http://catalogoviti.politicheagricole.it/catalogo.php) for nursery propagation that are widely used.

Although this large intra-varietal variability, the common fingerprinting methods based on simple sequence repeats (SSRs) markers cannot discriminate clones within ‘Nebbiolo’^25^. Because in recent years one of the most interesting and partially achieved objectives has been to distinguish clones within a grapevine cultivar using rapid and robust techniques, the genetic basis of phenotypic variation within ‘Nebbiolo’ was investigated via the sequencing of three ‘Nebbiolo’ certified clones: CVT 71, CVT 423, and CVT 185. The sequencing of ‘Nebbiolo’ clones and a *de novo* transcriptome assembly provided the dual purposes of: i) determining the genetic characteristics of ‘Nebbiolo’ using more than one clone, thus representing more accurately the genetic variability within this cultivar, and ii) identifying molecular markers able to discriminate these clonal selections.

## Results

### Genome sequencing and SNV identification in three ‘Nebbiolo’ clones

**Figure 1:**
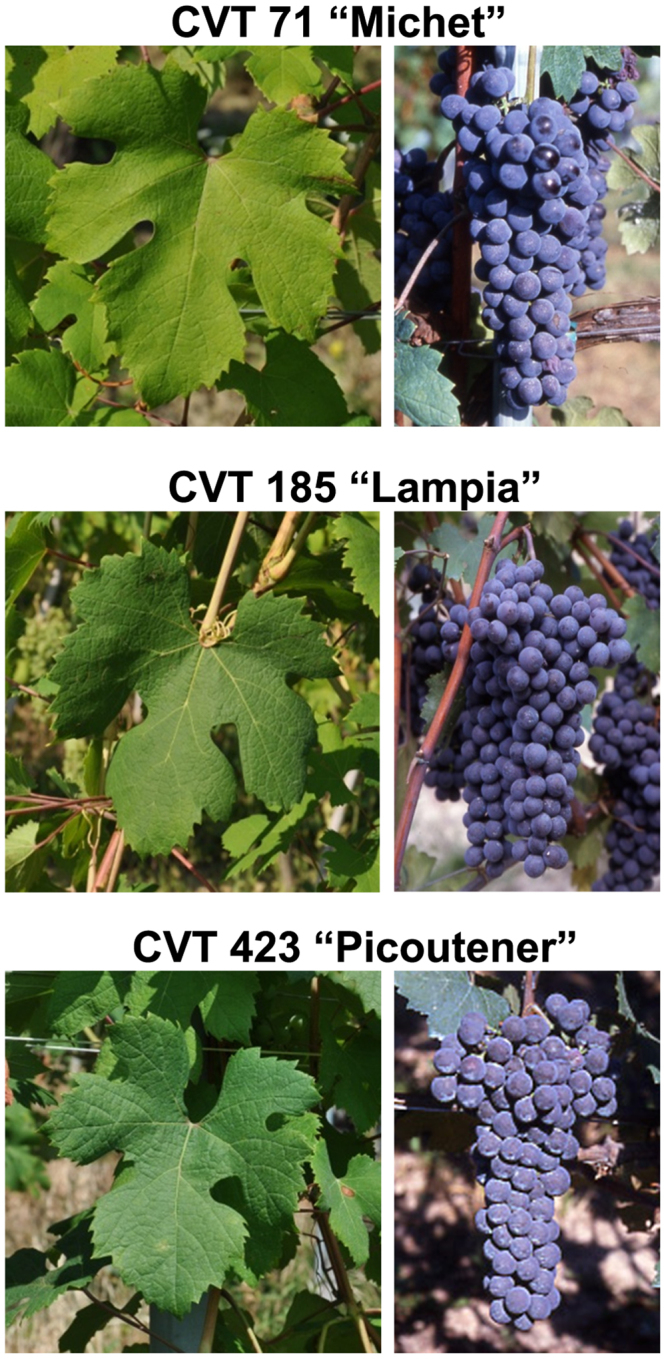
Phenotypic characteristics (leaves and bunches) of the three clones of ‘Nebbiolo’, CVT 185, CVT 71, and CVT 423, selected for genome sequencing.

The whole-genome sequencing of *V*. *vinifera* cv. Nebbiolo was performed on three clones of different geographical origins and phenotypic characteristics in order to provide an outline of the genetic intra-varietal variability of ‘Nebbiolo’. Clone CVT 71, which belongs to biotype “Michet”, is characterised by medium-high vegetative vigour and yield and good environmental stability. Clone CVT 185 is of biotype “Lampia” and was selected from vineyards located in southern Piedmont, as was CVT 71. CVT 185 is characterised by medium vegetative vigour, yield, and environmental stability. Clone CVT 423 (biotype “Picoutener”), which originated from the Aosta Valley, exhibits low vegetative vigour and yield and medium-low environmental stability (Fig. 1, Table S1).

Genomic DNA extracted from these three clones was used to generate between 99 million and 169 million Illumina reads (2 × 100) per clone, obtaining about 80 Gb of sequences representing as raw sequencing reads an average of 59-fold base coverage for of each clone (Table S2). After quality filtering, the average mapping rate of the reads in the grape reference genome PN40024 was approximately 95%, with an average of 117 million reads mapped uniquely per clone (Table S2). In order to genotype the three ‘Nebbiolo’ clones, SNVs identification was performed by comparing the aligned reads to PN40024, resulting in the identification of 7,207,952, 7,280,650, and 7,241,094 SNVs for CVT 71, CVT 423, and CVT 185, respectively. The GATK filtering procedure and the removal of variants located in repetitive regions resulted in a reduced set of 1,179,017 SNVs in the three clones. From these, (i) 503,648 SNVs were filtered-out using quality-based recalibration procedure based on validated SNVs, which is detailed in Material and Methods section, (ii) 6,571 SNVs were filtered-out because of presence of spurious alleles (i.e., SNVs for which more than two alleles are detected in at least one clone) and (iii) 3,291 were excluded from further analysis as they were located in regions not adequately covered in at least one of the three clones (Table 1). The final set of Nebbiolo variants comprised a total of 665,561 SNVs, substantially in line with the number of variants reported previously in the table grape ‘Sultanina’^10^.

### Transcriptome sequencing and assembly

**Figure 2:**
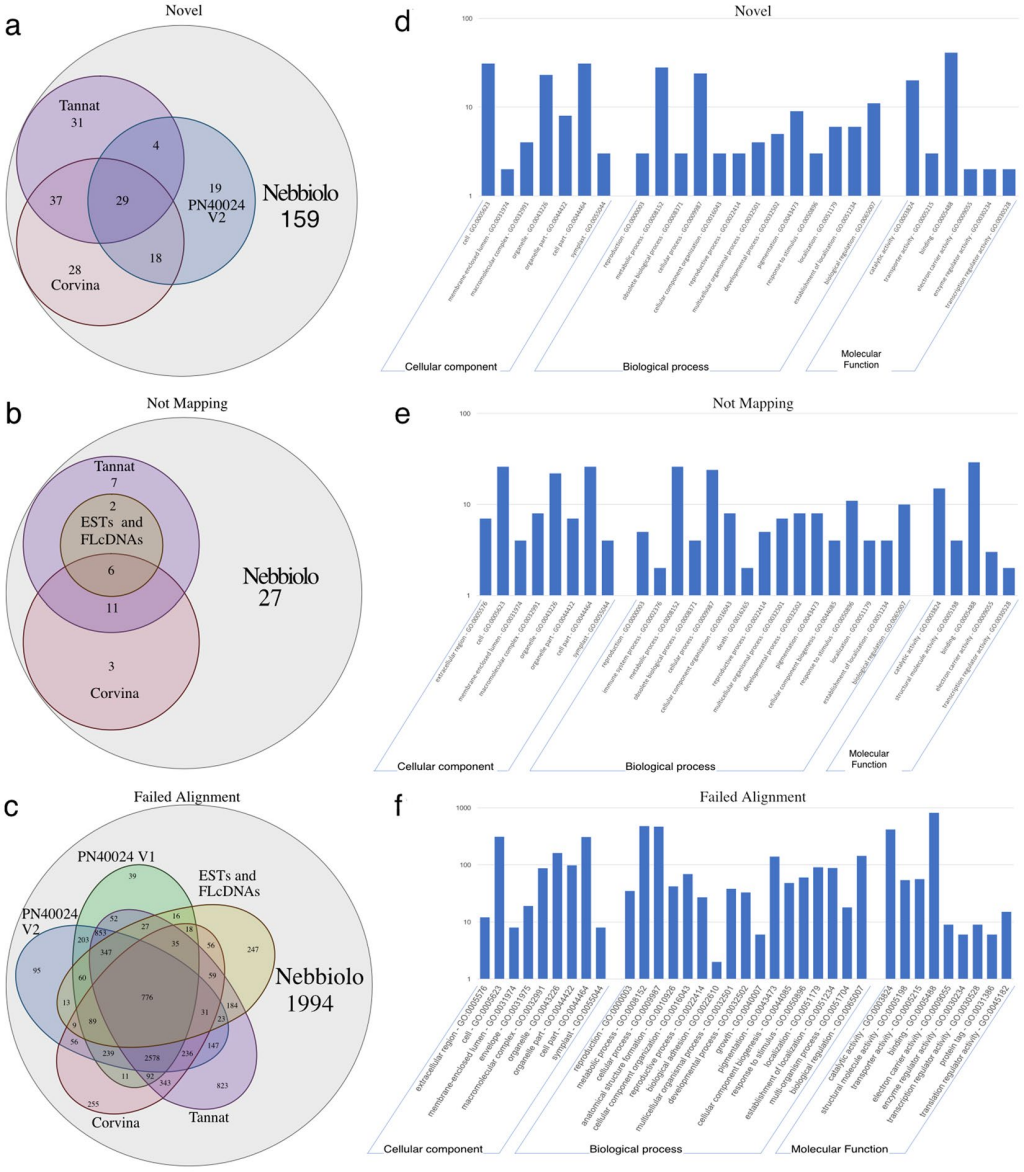
‘Nebbiolo’ CVT 71 transcriptome assembly comparison with other cultivar transcripts and ‘Nebbiolo’-specific transcript functional annotation. Panels (a), (b) and (c) show Venn diagrams of ‘Nebbiolo’ transcript matching with PN40024 V1 and V2 annotations, *Vitis vinifera* ESTs and the cDNA full-length database; ‘Tannat’ and ‘Corvina’ assembled transcriptomes, separated by aligning to the reference but not matching a known annotation (novel); or those assembled transcriptomes not aligning on the genome and with alignment rejected as failing quality control, respectively. Panels (d), (e) and (f) represent the same categories but with the distribution of GO terms for the putative ‘Nebbiolo’-specific transcripts.

For *de novo* transcriptome characterization of ‘Nebbiolo’, RNA-Seq analysis was performed using a variety of different tissues across several developmental stages collected from the clone CVT 71 (Table S3). A Duplex-Specific Thermostable Nuclease (DSN) normalised RNA-seq library was produced from a pool of 27 different tissues and sequenced, resulting in more than 208 million fragments (Table S4). Quality filtered reads were assembled *de novo*, producing 241,296 putative transcripts. After filtering, a final dataset of 44,961 putative protein-coding transcripts spanning more than 61 Mb was obtained, with an average length 1,357 bp (N50 length of 2,025 bp), which is in accordance with the PN40024 V1 gene annotation (N50 length of 1,755 bp). Of the 44,961 putative transcripts, 26,638 aligned to the genome and integrated into the PN40024 V1 gene annotation, enabling the update of 12,361 gene structures and the detection of 325 new unannotated loci. When comparing the new loci to the annotation of PN40024 reported by Vitulo *et al*.^4^ (referred to as V2) and to the ‘Tannat’ and ‘Corvina’ transcriptomes, we found that 159 of these loci were not shared with any other cultivar (Fig. 2b, Table S5). The putative protein-coding genes that could not be integrated into the PN40024 annotation were queried for non-Viridiplantae-associated-encoded proteins, and matching sequences were removed as putative contaminants. As a result, 56 putative transcripts that did not map in any region of the genome sequence were compared to *V*. *vinifera* ESTs and available full-length cDNA sequences and to the assembled transcriptomes of ‘Tannat’ and ‘Corvina’. As a result, 27 transcripts were defined as putative ‘Nebbiolo’-specific transcripts that were not shared with any other available cultivar (Fig. 2b, Table S5). The 10,107 filtered transcripts that failed to properly align with the genome were compared to all the available annotations of PN40024 (12x V1 and the V2 annotation^4^), to the available *V*. *vinifera* ESTs and full-length cDNAs, and to the ‘Tannat’ and ‘Corvina’ assembled transcriptomes. One thousand nine hundred ninety-four transcripts previously unidentified in grape cultivars were identified (Fig. 2c, Table S5). All the putative ‘Nebbiolo’-specific transcripts were functionally annotated; these transcripts covered a wide range of functions and act in different metabolic processes (Fig. 2d–f). Among the 1,994 putative ‘Nebbiolo’-specific transcripts that did not align with the genome, we observed as expected several not annotated transcripts (548) whose functions were not predictable. In addition, a surprisingly high number of genes, about one-quarter of the total (469), were involved in disease and stress resistance (Table S5). To validate the transcriptome data, 10 putative ‘Nebbiolo’-specific transcripts were assessed by end-point PCR and Sanger sequencing. Nine transcripts (90%) were efficiently amplified (Figure S1) from the pool of ‘Nebbiolo’ tissues (Table S3) used for RNA-Seq analysis, and the Sanger sequencing confirmed the sequences produced trough the transcriptome assembly. The transcripts were present also in another pool of ‘Nebbiolo’ tissues collected in 2017 from the clones CVT 185 and CVT 423 (Table S6). In addition no amplification was observed in similar tissues collected from PN40024 and ‘Barbera’ (Figure S1). ‘Barbera’ was used as example of another *V*. *vinifera* cultivar because it is cultivated in the same geographical area of ‘Nebbiolo’, while ‘Tannat’ and ‘Corvina’ are not present in the north-western Italy.

### Identification of SNVs shared by the three ‘Nebbiolo’ clones

**Figure 3:**
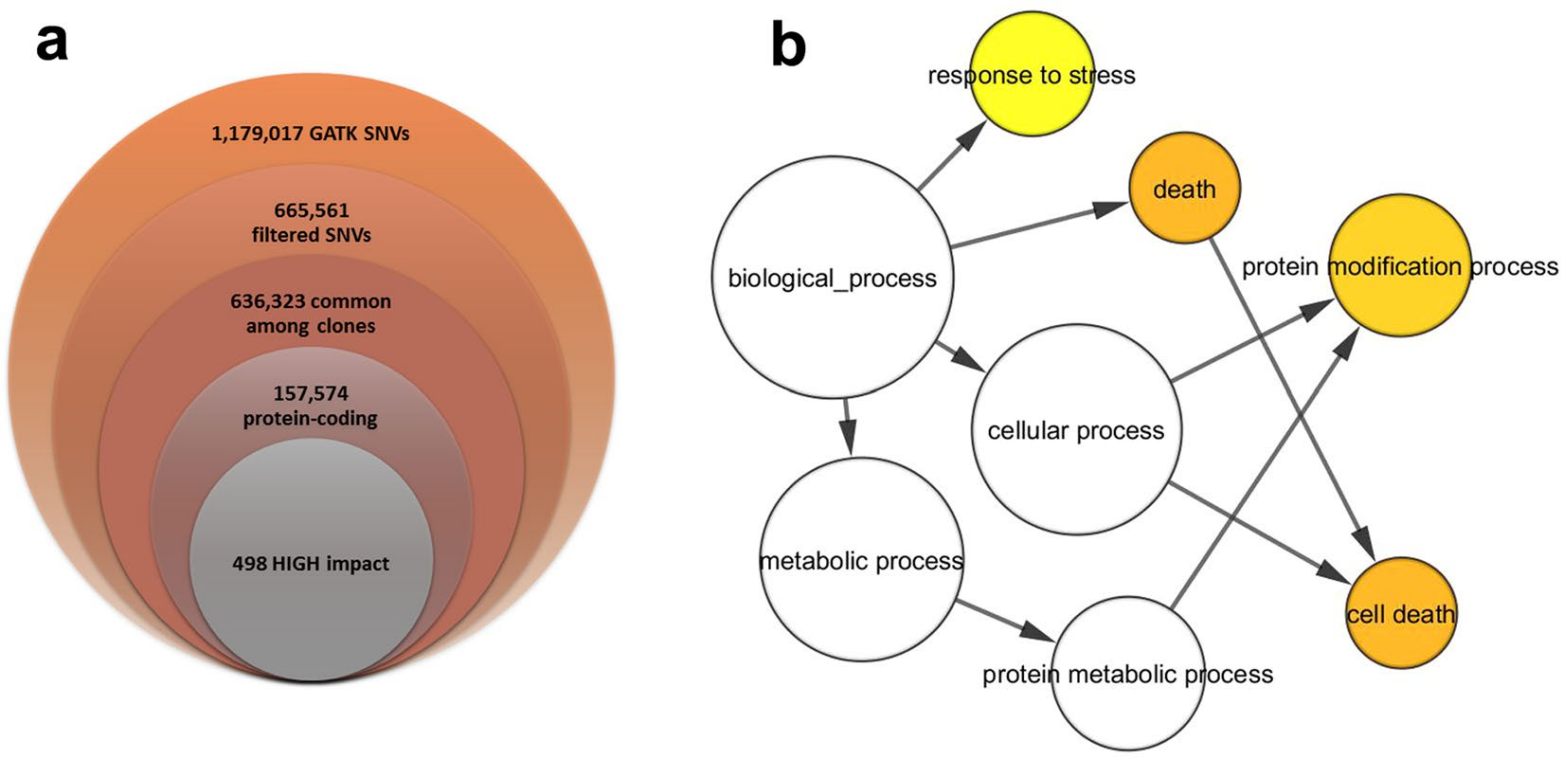
(**a**) The diagram shows the numbers of SNVs in common between the three ‘Nebbiolo’ clones and variant with respect to PN40024 reference genome among the total number obtained and the numbers of SNVs associated with coding regions having high impact on proteins. (**b**) BinGO results for over-represented GO biological processes of genes associated with high-impact putative ‘Nebbiolo’-specific SNVs (FDR < 0.05). The coloured circles indicate over-represented processes.

In order to identify SNVs shared by the three sequenced ‘Nebbiolo’ clones, we first searched for variants that showed the same genotype in all the clones, discarding those specific to a single clone. Among the whole set of called SNVs, 636,323 (95.6%) were shared by all three ‘Nebbiolo’ clones (Table 1), including 157,574 SNVs located in protein-coding regions, as defined by the V1 annotation of the PN40024 genome. As reported above for the putative varietal genes, several SNVs identified in ‘Nebbiolo’ were present also in the genomes of ‘Tannat’ and ‘Corvina’. Excluding these SNVs, we identified 5,458 genes containing SNVs that are not present in PN40024, ‘Tannat’, or ‘Corvina’ (Table S7). These genes were analysed by Gene Ontology (GO) enrichment analysis using BiNGO, a Cytoscape plug-in^26^. The processes of response to stress, cell death, and protein modification were significantly over-represented (Figure S2). The impact of the detected SNVs on the genes was classified according to SNPeff ver. 3.0 and is shown in Table 2. SNVs having a “high” impact included missense mutations or nonsense ones, such as the loss of the start or stop codon, the generation of a premature stop codon, or the alteration of splicing sites. The analysis showed that 455 genes were affected by at least one SNV common to the three ‘Nebbiolo’ clones (in total 498 SNVs with high impact) that contain potentially disruptive mutations (Fig. 3a). In this group of genes, the biological processes of response to stress and cell death were significantly over-represented (Fig. 3b). In addition, SNVs affecting 185 of these genes were absent in the ‘Corvina’ and ‘Tannat’ genomes (Table S8), and 34 showed SNVs also in the upstream region (5,000 nt upstream the transcription start site) of the coding sequences, mutations that could further affect their function (Table S8). Indeed, in addition to SNVs having a high impact on the genes, 32,659 SNVs were located in the upstream regions of 2,578 genes (Table S9) inducing potential changes in the regulation of the transcripts.

**Table 1:** Number of SNVs identified in the three sequenced ‘Nebbiolo’ clones divided into putative clone- specific and varietal SNVs. Clone-specific SNVs refer to SNVs with a genotype identified in one clone and with a different genotype in the other two clones. Putative varietal SNVs refer to SNVs present with the same genotype in all the clones.

Genotype	Putative clone-specific SNVs			Putative varietal SNVs	Total
	CVT 71	CVT 423	CVT 185		
**Homozygous reference**	784	403	400	—	1,587
**Homozygous alternate**	3,606	3,113	3,245	524,899	534,863
**Heterozygous**	8,496	4,398	4,425	111,424	128,743
**Excluded**	—	—	—	—	368
**TOTAL**	12,886	7,914	8,070	636,323	665,561

**Table 2:** Summary results of the SNPeff analysis of variant effects with respect to transcript structure, reported by category for the putative varietal ‘Nebbiolo’ SNVs. HETERO = heterozygous SNVs; HOMO ALT = homozygous alternate SNVs. Only high-impact SNVs were manually verified and selected for validation.

EFFECT	IMPACT	#HETERO	#HOMO ALT	#TOTAL
**START LOST**	HIGH MISSENSE	9	37	46
**STOP GAINED**	HIGH NONSENSE	54	164	218
**STOP LOST**	HIGH MISSENSE	5	83	88
**SPLICE SITE ACCEPTOR**	HIGH	15	62	77
**SPLICE SITE DONOR**	HIGH	15	54	69
**NON SYNONYMOUS START**	LOW MISSENSE	2	4	6
**SYNONYMOUS CODING**	LOW SILENT	3,473	11,630	15,103
**SYNONYMOUS STOP**	LOW SILENT	6	37	43
**START GAINED**	LOW	136	438	574
**NON SYNONYMOUS CODING**	MODERATE MISSENSE	3,651	13,125	16,776
**DOWNSTREAM**	MODIFIER	6,809	35,311	42,120
**INTRON**	MODIFIER	17,142	99,189	116,331
**UPSTREAM**	MODIFIER	5,196	27,463	32,659
**UTR 3 PRIME**	MODIFIER	789	4,639	5,428
**UTR 5 PRIME**	MODIFIER	799	2,163	2,962

**Figure 4:**
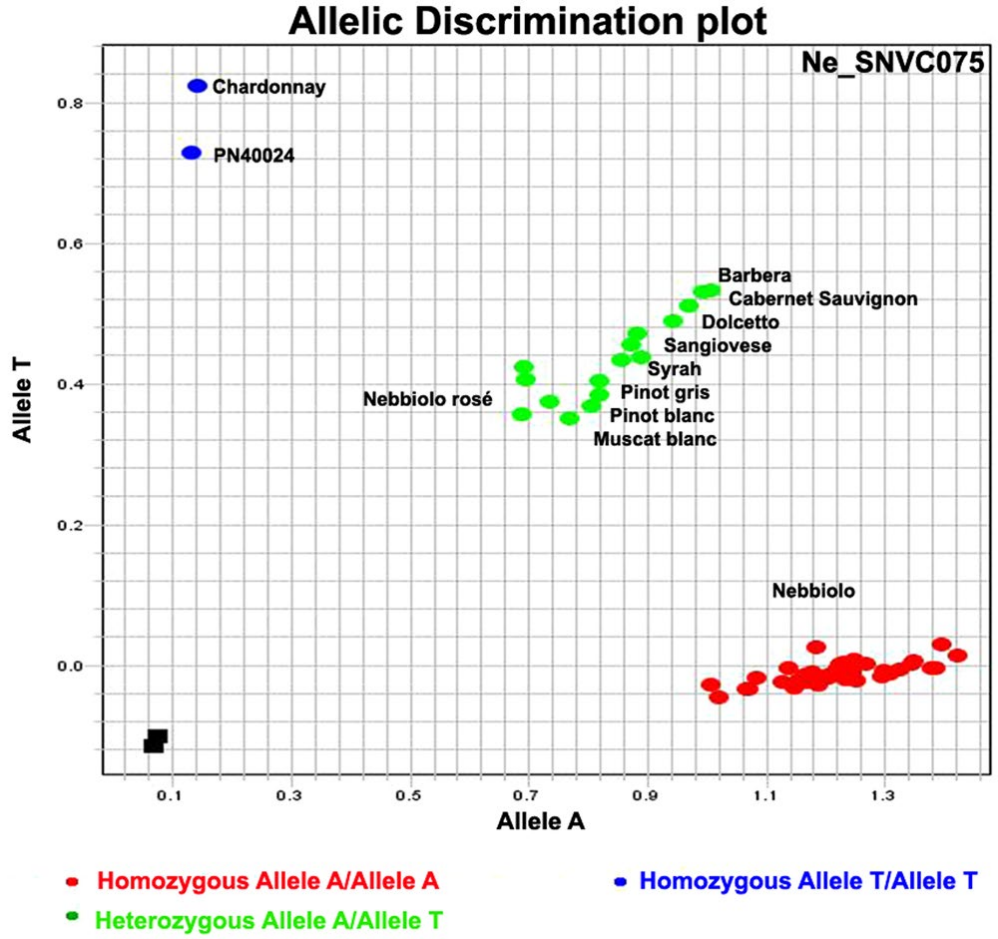
Output of TaqMan^®^ SNV genotyping assays for Ne_SNVC075, a putative SNV specific to ‘Nebbiolo’ clones. Each point is a clone of ‘Nebbiolo’ or another *V*. *vinifera* cultivar.

To validate some SNVs derived from Illumina data, 10 SNVs common to the three clones having a high impact on the protein-coding portion of the genes were assessed by Sanger sequencing. Nine SNVs (90%) were confirmed (Table S10), and for one of them (Ne_SNVC075, associated with the disease resistance gene VIT_18s0001g03900), it was possible to design TaqMan^®^ probes for genotyping assays. The allelic combination for the Ne_SNVC075 observed in the three sequenced ‘Nebbiolo’ clones (A/A) was confirmed in several ‘Nebbiolo’ clones analysed (Table S11). In addition, different allelic combinations for this SNV were observed in the sets of different international, Italian, and local grapevine cultivars. In particular, the heterozygous combination A/T was present in ‘Nebbiolo rosè’, a genotype related to ‘Nebbiolo’ by kinship, ‘Barbera’, ‘Cabernet Sauvignon’, ‘Dolcetto’, ‘Lambrusco Montericco’, ‘Malvasia Nera’, ‘Muscat blanc’, ‘Pinot blanc’, ‘Pinot gris’, ‘Sangiovese’ and ‘Syrah’. The alternative homozygous combination T/T was present in ‘Brachetto’, ‘Chardonnay’, ‘Gaglioppo’, ‘Merlot’ and PN40024 (Fig. 4).

### Putative ‘Nebbiolo’ clone-specific SNVs

In addition to SNVs shared among the three clones, 28,870 SNVs showed a specific genotype in one of the three ‘Nebbiolo’ clones (Table 1). We retained SNVs having a particular genotype in one clone and a different genotype in the other two clones. A set of 368 SNVs having different genotype in all three clones was not considered for further analyses (Table 1). 2,751 SNVs from CVT 185, 4,803 from CVT 71, and 2,606 from CVT 423 were located in protein-coding regions defined by the V1 annotation of the PN40024 genome (Figure S3). The analysis showed that 2,665 genes were affected by at least one SNV specific to CVT 71, 1,743 genes were affected by SNVs specific to CVT 185, and 1669 genes by SNVs specific to CVT 423. A total of 21 genes were predicted to have potentially disruptive mutations (Table S12).

The putative clone-specific SNVs were further analysed to identify markers useful for clonal identification. To this purpose, first, SNVs located in repetitive regions and low-confidence SNVs were removed from the whole list and underwent specific filtering procedure to select candidates for validation (see Materials and Methods). Second, the remaining SNVs were reviewed by analysing the 300–500-bp region including the SNV and excluding the SNV when the interested region had multiple hits on the genome. Third, the feasibility of designing TaqMan^®^ probes in correspondence of the mutation was evaluated. After validation by Sanger sequencing, five SNVs specific to clone CVT 423, three SNVs specific to CVT 185, and three to CVT 71 (Table S10) were found. The results obtained from TaqMan^®^ SNV genotyping were generally consistent with the Sanger analysis. However, Ne_SNV45 showed problems in the amplification and was excluded from further analyses, and for Ne_SNV31, Ne_SNV33 and Ne_SNV62, the allelic calls for clone CVT 185 showed some discrepancies in comparison to sequencing data (Figure S4). In order to deepen these inconsistences and to verify the presence of periclinal chimeras, in the three sequenced clones we amplified by TaqMan^®^ genotyping assay the 10 validated clone-specific SNVs in berry skin and berry flesh extracted separately. The genetic profile of layer L1 was deduced from the difference between leaf and berry skin (derived from both cell layers L1 and L2) and berry flesh (originated only from L2 layer) profiles. For seven SNVs no difference was observed in the allelic calls between berry skin and berry flesh in all the three sequenced clones. Conversely, the clone CVT 185 in correspondence to Ne_SNV31, Ne_SNV33 and Ne_SNV62 showed clearly different allelic calls in berry skin/leaf and berry flesh (Figure S4). For the three loci the homozygous condition reported in Table S10 was confirmed only in berry flesh, while in the allelic discrimination plots the berry skin/leaf were located in a position between heterozygous and homozygous conditions (Figure S4). This can suggest a chimerism and different genetic profiles between L1 and L2, likely homozygous or hemizygous in L2 and heterozygous in L1. For example, for the locus Ne_SNV31, the genetic profile of clone CVT 185 was C/C or hemizygous C for the L2, and C/T in the L1, consequently in leaf and berry skin the allelic call was positioned in an intermediate position because the frequency of allele T was lower than in a classic heterozygous (Figure S4). These results were confirmed by Sanger sequencing after cloning the fragments amplified from berry skin and berry pulp. Only the allele C was detected in berry pulp of CVT 185, while in berry skin the allele T was detected in one colony out of 5, confirming a chimerism between L1 and L2.

The 10 SNV assays were then tested on 98 ‘Nebbiolo’ accessions collected from typical geographical areas of ‘Nebbiolo’ cultivation: southern Piedmont (Langhe and Roero), the Turin hillside, northern Piedmont (Canavese and High Piedmont, consisting in an area between the town of Biella and the Ticino River, Ossola Valley), the lower Aosta Valley, and northern Lombardy (Valtellina) (Table S11). Among these areas, 40 accessions consisted of clones included in the Italian National Register of Grape Varieties, selected from mother plants scattered in the typical areas of ‘Nebbiolo’ cultivation. The remaining 58 samples were collected from old plants (aged no less than 70–80 years) and did not undergo the clonal selection process. The goal of this survey was to cover most of the areas where ‘Nebbiolo’ was and is currently present, analysing the most ancient plants typical of each area. Both registered clones and ancient local vines should therefore be considered putative unique accessions, henceforth referred to simply as clones. Samples were then genotyped at 6 SSR loci indicated as molecular descriptors for grape varietal identification^27^, and the results confirmed that all selected accessions were the true-to-type Nebbiolo cultivar.

**Figure 5:**
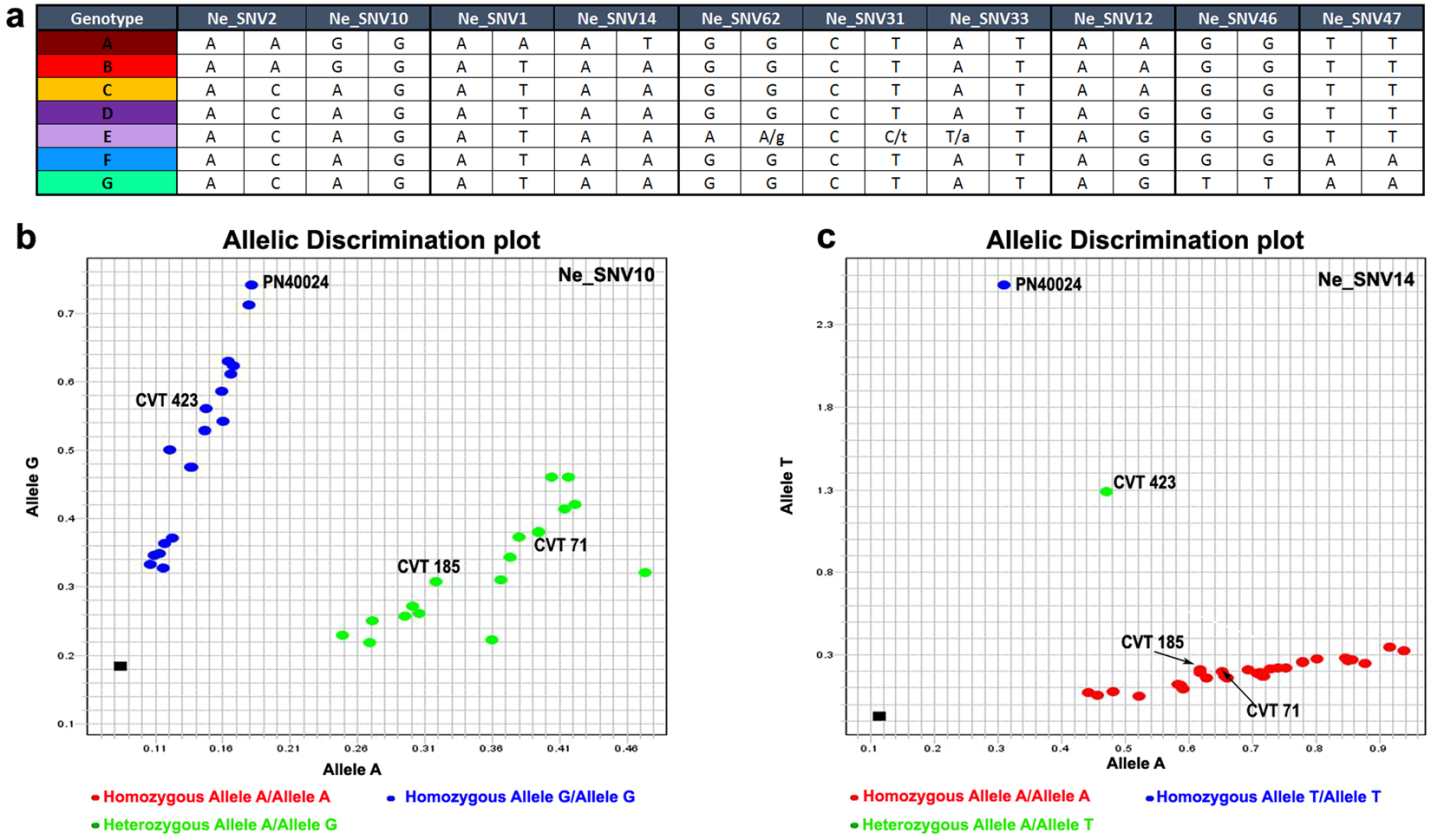
(**a**) Summary table of the genetic profiles of the seven ‘Nebbiolo’ genotypes (from A to G) obtained by combining the outputs of the TaqMan^®^ genotyping assays specific to 10 clonal SNVs. The lowercase letters indicate the likely presence of periclinal chimeras. Example of the output of TaqMan^®^ SNV genotyping assays specific to (**b**) Ne_SNV10 and (**c**) Ne_SNV14.

The 98 ‘Nebbiolo’ clones were grouped into seven genotypes (from A to G) based on the analysis of 10 SNV markers, and in particular, only six SNVs could sufficiently discriminate the seven genotypes (Fig. 5, Table S11). Each genotype comprised a different number of samples, ranging from 31 for genotypes B and D to a single clone for genotype A. Ne_SNV1 and Ne_SNV14 were specific markers for genotype A (clone CVT 423): one of them was sufficient for distinguishing this clone from all others (Fig. 5, Table S11). Similarly, only one SNV among Ne_SNV31, Ne_SNV33, and Ne_SNV62 was sufficient for identifying the CVT 185 and CVT 180 clones (genotype E). The chimerism identified in CVT 185 (Figure S4) was confirmed for all three SNVs also in the clone CVT 180, suggesting a close genetic relationship between these two clones. The two clones belonging to genotype C were uniquely identified using Ne_SNV2 and Ne_SNV12 (Fig. 5, Table S11).

**Figure 6:**
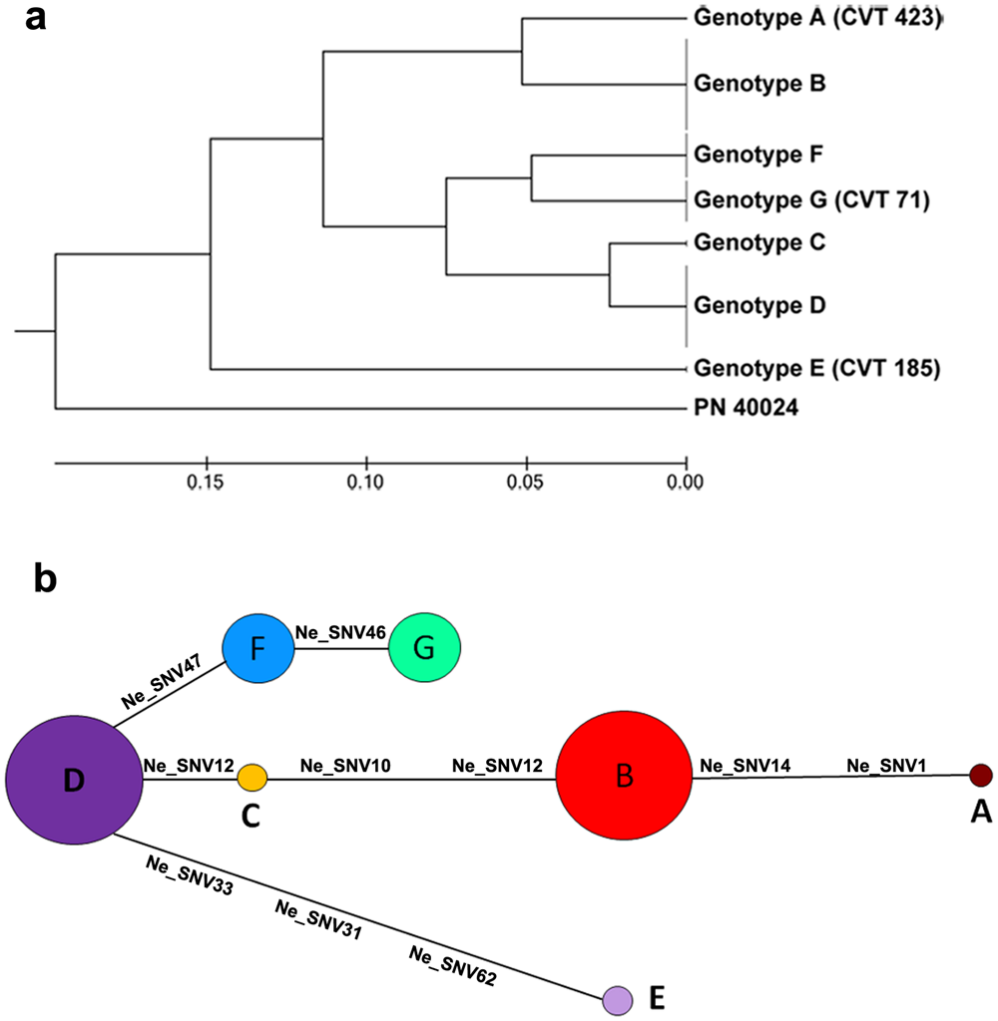
(**a**) The neighbour-joining tree of the seven genotypes of ‘Nebbiolo’ and the PN40024 reference genome. The significance of each node was tested using 1,000 bootstrap replicates. (**b**) Median network representing all genotypes identified in ‘Nebbiolo’. Circled areas are proportional to genotype frequencies in the global sample.

The genetic relationships among ‘Nebbiolo’ genotypes were examined using both an unweighted pair-group method using arithmetic average (UPGMA) dendrogram and a median network diagram by two cluster analyses (Fig. 6). In the dendrogram (Fig. 6a), the genotypes clustered into three groups: A and B merged into one cluster, whereas C, D, F, and G were separated into two subgroups belonging to a second cluster. Another cluster included only genotype E, and the reference genome (PN40024) was clearly separated from the ‘Nebbiolo’ genotypes. In the median network, each genotype constituted a separate group (Fig. 6b). The results showed that the most frequent genotypes (B and D) corresponded to two major lineages. Genotype D occupied a central position and was closely associated with genotype F, from which genotype G is derived; and genotype C, which represents the link between genotypes D and B. Genotype E was likely a periclinal chimera originated from Genotype D, as well as the genotype A (CVT 423) was likely generated through mutations in genotype B (Fig. 6b).

**Figure 7:**
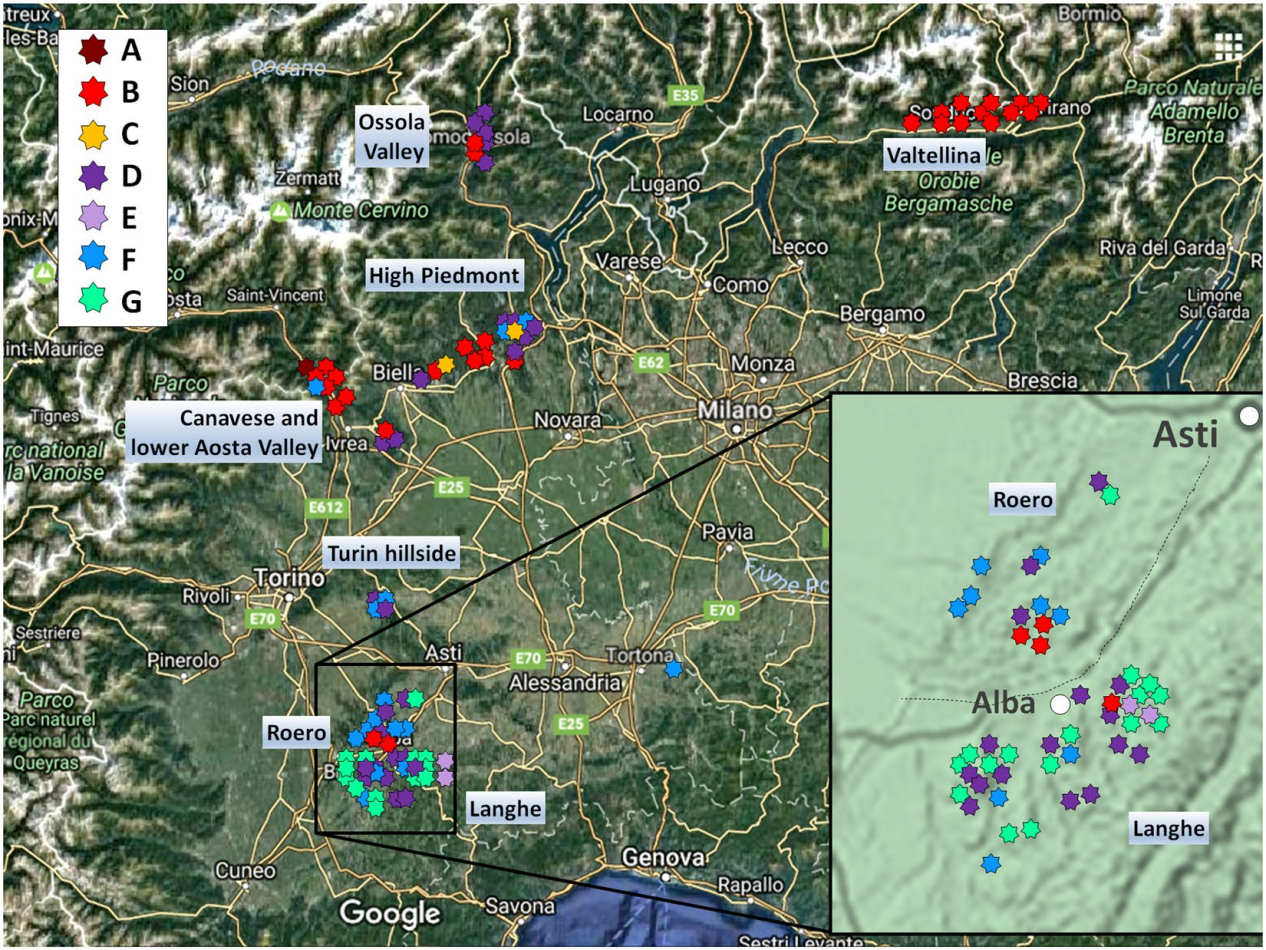
Geographical distribution in north-western Italy of the 98 analysed ‘Nebbiolo’ clones classified according to the seven genotypes identified using SNV markers. Map modified from: Immagini©2017 Landsat/Copernicus, Data SIO, NOAA, U.S. Navy, NGA, GEBCO, Dati cartografici ©2017 GeoBasis-DE/BKG (©2009), Google. (https://www.google.it/maps/@45.2990884,7.1806928,294174m/data=!3m1!1e3).

A clear distribution of the certified clones (reported in grey in Table S11) according to their geographic origin was observed for the first time: the clones from northern Piedmont and Lombardy (Valtellina) belonged to the genotype A and B, whereas those from southern Piedmont (Langhe and Roero) showed the genetic profiles of genotypes D, E, F, and G. When considering the whole set of samples and examining the geographical distribution of the genotypes, it was evident that some prevailed in certain areas and some were absent in others (Fig. 7). Genotype B was more frequent in northern Piedmont, and interestingly, it was the only genotype in Valtellina (Fig. 7). Genotype D was well distributed throughout all areas (except Valtellina), whereas G and E were typical of southern Piedmont and, in particular, Langhe (Fig. 7).

## Discussion

The high complexity of plant genomes, including the extent of repetitive content and high heterozygosity rates in diploid and polyploid genomes, make the *de novo* assembly of genomes from short-read sequencing data challenging^7^, and *V*. *vinifera* cv. Nebbiolo is no exception. Grapevine haplotypes can differ by the presence of extended structural variations^8^ that leads to extreme sequence divergence and heterozygosity. ‘Pinot Noir’ PN40024 used for the *V*. *vinifera* reference assembly was specifically bred to be a highly homozygous variety, which lowered the heterozygosity rate to 7%^1^, facilitating the sequence reconstruction process. Although the detection of short variations in the genome sequence led to the underestimation of ‘Nebbiolo’ heterozygosity (1.2%), values were higher than those reported for the genomes of ‘Tannat’^5^ and ‘Thompson seedless’^10^, suggesting higher levels of mutations, heterozygosity, and hypothetically higher diversity between parental haplotypes in ‘Nebbiolo’.

One of our main objectives, which was made possible via the sequencing of three ‘Nebbiolo’ clones and the *de novo* assembly of the transcriptome of one ‘Nebbiolo’ clone, was to determine the genetic characteristics typical of the ‘Nebbiolo’ variety in order to identify ‘specific’ mutations and genes of this cultivar, taking into account the intra-varietal variability.

The comparison of the transcripts assembled for ‘Nebbiolo’ CVT 71 with the PN40024 V1 and V2 genome annotations allowed the identification of 159 novel loci expressed in ‘Nebbiolo’ but not previously annotated in grape. There were 2,180 transcripts not shared with any other previously annotated cultivar, as no representative could be found in PN40024 gene space or in ‘Tannat’ and ‘Corvina’ transcriptomes. These transcripts were new discoveries and could be considered specific to the ‘Nebbiolo’ cultivar. Nevertheless, the definitions of ‘Nebbiolo-specific’ or ‘varietal genes’ were used in this context only to simplify the concept, as there are thousands of different cultivars of grapevine spread throughout the world, and these transcripts could theoretically be found in other cultivars in addition to ‘Nebbiolo’. Indeed, the comparison with ‘Tannat’ and ‘Corvina’ clearly showed that cultivar-specific genes should be reconsidered every time the transcriptome of a different variety is produced. The validation by Sanger sequencing of nine novel transcripts (only for one transcript we did not observe any amplification in ‘Nebbiolo’) confirmed the high level of reliability of the transcriptome assembly. In addition, the expression of these genes in ‘Nebbiolo’ tissues collected in different vineyards, clones and years, and at the same time the absence in PN40024 and in ‘Barbera’ cultivated near to ‘Nebbiolo’ in the same vineyard (Figure S1), support the considerations about the peculiarity of ‘Nebbiolo’ transcriptome. Among the putative ‘varietal’ transcripts of ‘Nebbiolo’, we observed the over-representation of genes involved in disease resistance (Table S5). These results suggested that the ‘Nebbiolo’ genome has a greater availability of resistance genes than do the reference genome and the ‘Tannat’ and ‘Corvina’ assembled transcriptomes.

To characterise the ‘Nebbiolo’ genome, we essentially focussed on SNV identification, as short-read sequencing in some case has not been proven to be reliable in capturing the complex structural differences characterising the different grape cultivars^5,8^. Considering the SNVs shared among the three ‘Nebbiolo’ clones, excluding the point mutations retrieved in the genomes of ‘Corvina’ and ‘Tannat’, we identified 6,262 genes potentially influenced by putative ‘Nebbiolo’-specific mutations. As reported above for the ‘Nebbiolo’ transcripts, also the definition of putative ‘Nebbiolo-specific’ SNVs was used in this context only to simplify the concept, as these SNVs could theoretically be found in other cultivars in addition to ‘Nebbiolo’. However, expanding on the analysis of one of these SNVs (e.g., Ne_SNVC075, Fig. 4), we have demonstrated that this locus, which is shared among the three sequenced clones, is common to all clones (98) collected in the typical geographical areas of the cultivation of ‘Nebbiolo’, but is polymorphic in 16 international and local cultivars. Thus, the sequencing of several clones of the same cultivar that are phenotypically different proved to be a powerful approach and likely produced better results than did the sequencing of a single accession in terms of identifying molecular markers typical of a specific cultivar. The ‘Nebbiolo’-specific SNV markers identified in this study could integrate into the existing dataset, implementing the genotyping approaches^28–31^. Another interesting aspect was linked to the associations among putative ‘Nebbiolo’-specific SNVs and genes that induce potential changes in the functionality (mutations in splicing sites, start lost, stop gained, etc.) and in the levels of gene transcription (mutations in the promoters)^32^. For instance, many genes associated with SNVs belong to specific functional categories, in particular, responses to pathogens (Fig. 3b). Similar enrichment in specific GO categories of mutations and/or varietal genes was observed in ‘Nebbiolo’ ‘varietal’ genes reported in this study (Table S5, Figure S2), and previously in other grapevine genomes^5,6,10^ and in other species^33,34^. In those cases, the authors explained the phenomenon as the result of a combination of effects derived from natural and artificial pressure selections. Specifically, the mutations in resistance genes and the ‘Nebbiolo’ ‘varietal’ genes likely reflect the environment in which ‘Nebbiolo’ was cultivated and to which it became adapted throughout hundreds of years of pressure from environmental stresses. Several of these genes (Table S5 and S7) belong to NB-LRR class involved in the resistance and/or tolerance to many pathogens^35^. Although the response of a plant to stresses is determined by many factors and the Nebbiolo cultivar cannot be defined as resistant to biotic stresses, it should be noticed that, for example, ‘Nebbiolo’ is less susceptible to Flavescence dorée phytoplasma (FDp) than other cultivars such as ‘Barbera’ and ‘Pinot’, showing low FDp titres and less symptoms^36,37^. However, the presence of these mutations could suggest in addition a deficiency in the reference genome, particularly with respect to these genes. Indeed, the reference genome showed some limitations, as PN40024 was essentially derived only from two cultivars (‘Pinot noir’ and ‘Helfensteiner’) and several cycles of self-fertilisation, which was undertaken to increase its level of homozygosity^1^; this phenomenon probably led to the loss of particular allelic variants. The resistance to stress may be among the most polymorphic and variable functional categories in grapevine and is probably under-represented in the reference genome. This further strengthens the awareness that the reference genome does not cover all the genetic variability present not only within the genus *Vitis* but also within the *V*. *vinifera* species^4^.

The second main objective of this work was to deepen the intra-varietal variability present in ‘Nebbiolo’, focussing on molecular markers able to identify three sequenced clones. In recent years, the demand for effective and efficient clonal identification tools that can ensure a tracking system for the propagated material, the ability for clones to be patented and a more efficient management of the germplasm has been increasing. However, the discrimination among clones is often extremely difficult. SSRs, the most common molecular markers used in grape varietal fingerprinting^27^, have rarely been observed among different clones of a cultivar^17,38–40^. Other molecular markers have been applied for distinguishing grapevine clones, such as inter-single sequence repeat (ISSR), amplified fragment length polymorphism (AFLP), selective amplification of microsatellite polymorphic loci (SAMPL), microsatellites amplified fragment length polymorphism (M-AFLP)^41–43^, and methylation-sensitive amplified polymorphism (MSAP)^44^. Although some of these methods seemed to be effective at identifying intra-varietal polymorphisms, they were generally laborious, expensive, time consuming, and difficult to employ in laboratories with low specialisation. Therefore, we focussed our analyses on the identification of SNV markers and on the development of an effective SNV genotyping system that is fast and easily exportable to all laboratories. Starting from the 28,870 SNVs specific to one of the three sequenced ‘Nebbiolo’ clones, we selected SNVs located in genomic regions where TaqMan^®^ genotyping assays were applicable. The Sanger sequencing showed a rate of confirmation of 90% for SNVs common to three clones, while for putative clone-specific SNVs the percentage was around 61%. The majority of the non-confirmed SNVs were predicted to be heterozygous in one clone, but after Sanger sequencing, they resulted homozygous concordant with the other two clones (Table S10). This could be ultimately due to errors in the PCR amplification step before Sanger sequencing with possible allelic drop out, as suggested previously in table grape^11^. In addition, as reported for clone CVT 185 possible chimerisms could hinder the analyses. This problem emerged clearly in the clonal-specific SNVs, as at least one of the three clones was frequently heterozygous at a single locus that was polymorphic among the three clones, whereas the putative ‘Nebbiolo’-specific SNVs associated with coding sequences were frequently homozygous alternatives to the reference genome.

The data relative to Nebbiolo clone-specific SNVs provided interesting information regarding this cultivar with respect to similar data previously published. For example, Carrier *et al*.^9^ reported a low polymorphism rate among three ‘Pinot noir’ re-sequenced clones, detecting only 19 SNVs. This small number of identified SNVs (1.6 SNVs per Mb), in comparison to a total of 28,870 Nebbiolo clone-specific SNVs, can be partially linked to the closer genetic relationship either among the three ‘Pinot noir’ clones and between the clones and the reference genome PN40024, and also likely derived by the low sequencing coverage. Indeed, the sum of the sequences shared by one of the three ‘Pinot’ clones and reference genome represents only 4.5 Mb (around 1% of grape genome) at 6-fold genome coverage^9^. A total of 14 SNVs instead were identified between white wine Spanish cultivar ‘Pedro Ximenes’ and the ‘Corinto bianco’, its parthenocarpic seedless somatic variant, starting from RNA-seq data of flower buds^45^. Despite the low level of polymorphism between these two somatic variants, these SNVs resulted particularly interesting because involving genes potentially responsible for parthenocarpic phenotype^45^. Results comparable with ‘Nebbiolo’ data have been obtained after the sequencing of four ‘Pinot’ somatic variants and two ‘Sangiovese’ clones^46^. In ‘Nebbiolo’, by comparing the aligned reads to PN40024, more than 7,200,000 SNVs have been identified in the three clones, while only 4,600,000 in ‘Pinot blanc’ and ‘Pinot Meunier’, and about 6,000,000 in ‘Sangiovese’ clones^46^. These data suggest that ‘Nebbiolo’ and ‘Sangiovese’ are genetically more distant to PN40024 than the ‘Pinot blanc’ or ‘Pinot Meunier’, and likely the higher average coverage of ‘Nebbiolo’ and ‘Sangiovese’^46^ genomes could influence positively the number of SNVs identified. In addition, on 55 SNVs detected between ‘Sangiovese’ clones after reads filtering, only three were validated, suggesting a close relationship between the two clones^46^. On the contrary, our initial choice of three phenotypically divergent ‘Nebbiolo’ clones (Table S1) results also in high genetic polymorphism rate, with a number of clone-specific SNVs ranging from 7,914 to 12,886 for a clone (Table 1), and with a rate of SNVs experimental validation of 61%. In summary, our results confirm the presence of an extensive inter and intra-varietal heterogeneity among the genomes of the different grapevine cultivars^5,6,9,28,46^, as also reported in other plant species^47^.

We identified and developed TaqMan^®^ genotyping assays based on real-time amplification for 10 SNVs. Thus, by exploiting this methodology, we extended the analysis to 98 different ‘Nebbiolo’ clones collected from typical regions of cultivation, and for the first time, the ‘Nebbiolo’ clones were classified using molecular markers in seven groups or genotypes (Table S11). Interestingly, Ne_SNV1 and Ne_SNV14 were specific markers for CVT 423 (genotype A), and it was possible distinguish quickly and certainly a single and registered ‘Nebbiolo’ clone from all others.

These results suggest SNV analysis is a promising and easy-to-use resource for the identification of molecular markers for clonal identification and could be ideally performed for all clones of each cultivar, starting from partial or whole-genome sequencing data. We hope this pioneering study will pave the way towards whole grapevine clonal identification, not only for distinguishing materials at the nursery level but also for legally protecting and patenting the selections of unquestionable identity. In addition, the sensitivity of TaqMan^®^ genotyping assay has also been useful in identifying periclinal chimeras, as demonstrated for chimerism associated to Ne_SNV31, Ne_SNV33, and Ne_SNV62 in the genotype E (clones CVT 185 and CVT 180). In the other clones analysed we did not observe any ‘unconventional’ allelic calls as reported for CVT 185 (Figure S4); however, we cannot rule out that other forms of mutations or hemizygous phenomena can be associated to these 10 SNVs in some of these clones. Indeed, in some variants that imply a passage from heterozygous to homozygous condition (Fig. 5), it cannot be excluded that these changes were the result of hemizygous deletions from the ancestral genotype rather than of single nucleotide mutations. Therefore, at least two ‘Nebbiolo’ clones are likely affected by the phenomenon of chimerism, as the well-known mutations associated to berry colour in several cultivars^18,19,48,49^.

The ability of clones to evolve is a critical concern for wine growers who use vegetative propagation to perpetuate virtually identical clones over time. The range of clonal diversity would depend on the age of the variety; the more ancient the variety, the longer it would have been exposed to environmental stresses and the longer it would have been accumulating mutations. Moreover, high clonal diversity may reflect the long-term interest of vine growers and winemakers to select clones with particular characteristics^17^. Then, clonal-specific SNVs were used to study the genetic relationships among the seven identified ‘Nebbiolo’ genotypes, which provided insight into the history of ‘Nebbiolo’ and its spread. The likely network model of genotype relationships (Fig. 6b) suggests that genotype D could be the ancestral genotype of the Nebbiolo cultivar from which the others originated through successive somatic mutations (Fig. 6b). Indeed, genotype D was not only abundant but also the most widespread profile in the cultivation areas of ‘Nebbiolo’, with the exception of Valtellina (Lombardy) (Fig. 6). ‘Nebbiolo’ could therefore have arisen in any area of Piedmont. The earliest historical quotations all refer to this region and were coeval in different areas: the Turin surroundings (1266), Alba district (1287), Asti area (1295), Roero (1303), and the Ossola Valley (1309)^20,22^. In Valtellina, only genotype B was present, although the vines investigated in this area were scattered throughout the whole valley. This could suggest a relatively late introduction of ‘Nebbiolo’ to the area (with a consequent limited time for diversification) and/or the introduction of a single genotype. The early quotations of ‘Nebbiolo’ in Valtellina date back to the end of the sixteenth century^23^, but were reported three centuries earlier in Piedmont. In northern Piedmont (Canavese and High Piedmont) and the lower Aosta Valley, in addition to the likely progenitor genotype D, other genotypes such as F and C were also present; these genotypes showed a central position in the network (Fig. 6b) and were closely related to D. Although considering D, F, and C the more ancient genotypes was precarious, it questions whether the cradle of ‘Nebbiolo’ was this part of Piedmont. In addition, since the end of the XIX century the changes consequent to the phylloxera crisis and to clonal selection programs could have greatly affected the spread of different genotype of grapevine and ‘Nebbiolo’. A much larger set of genetic markers and old mother plants from all areas where ‘Nebbiolo’ has been cultivated throughout the centuries must be investigated in order to confirm this still weak albeit fascinating hypothesis.

In conclusion, the sequencing of three ‘Nebbiolo’ clones has provided interesting insights about the genetic peculiarities of this cultivar and its intra-varietal variability, which is useful for clonal identification. The sequencing of multiple clones for a single cultivar is an innovative approach, and for the first time, these analyses have been exploited to develop an efficient and reliable method for clonal identification in grapevine. The knowledge of the genetic basis of multiple variants present in grapevine associated with functional genomics studies will likely be implemented into classical breeding methods and would be fundamental for large-scale applications of the new ‘sustainable biotechnologies’ (i.e., *cis*-genesis and genome editing in grapevine)^50–52^.

## Material and methods

### Plant material and nucleic acid extraction

Three clones of *V*. *vinifera* ‘Nebbiolo’ (CVT 71, CVT 423, and CVT 185) registered in the Italian National Register of Grape Varieties by the Institute for Sustainable Plant Protection, National Research Council (IPSP-CNR) were selected for whole-genome sequencing (Fig. 1). Young leaves were collected from the primary source plants of each clone conserved in a dedicated screenhouse in Alba (Cuneo, Italy). Genomic DNA was extracted in accordance with the protocol of Carrier *et al*.^53^ that was developed for the extraction of high-quality DNA with a low level of cytoplasmic DNA contamination.

Samples for RNA library production were collected in 2013 from the plants of ‘Nebbiolo’ clone CVT 71 in a commercial vineyard located in Monforte d’Alba (Cuneo, Italy). Plants were trained to a vertical trellis using Guyot pruning; conventional agronomic management was regularly applied in the vineyard. Twenty-seven samples were collected from different organs and at different phenological phases according to the E-L system modified by Coombe *et al*.^54^, as reported in Table S3. The roots were collected from 3-year-old greenhouse-grown potted plants. For each sampling, materials from at least 10 plants were pooled, immediately frozen in liquid nitrogen and stored at −80 °C. In addition to the material above described, for the validation of putative ‘Nebbiolo’-specific transcripts, different organs were collected in 2017 from plants of ‘Nebbiolo’ clones CVT 185 and CVT 423, PN40024 and ‘Barbera’ (Table S6) cultivated in a vineyard located in Grugliasco (Torino, Italy). Total RNA was extracted using the Spectrum™ Plant Total RNA extraction kit (Sigma Aldrich) starting from 100 mg of plant material, and RNA quantity was verified using a NanoDrop 1000 spectrophotometer (Thermo Fisher Scientific). RNA quality was verified on an RNA 6000 Nano Labchip using a Bioanalyzer 1000 (Agilent Technologies, Santa Clara, CA, USA); all samples had RIN values ≥7.

### Library preparation, sequencing, and data pre-processing

Genomic DNA (1.5 μg of each clone) was sonicated for 115 s using a Covaris S2 instrument (Covaris, Inc., Woburn, MA, USA) and purified using Agencourt AMPure XP beads (Beckman Coulter, Krefeld, Germany) in two steps (0.45x and 1.3x) in order to obtain fragments ranging from 200 bp to 800 bp in length. The quality of the fragmented DNA was determined using an Agilent DNA 1000 kit (Agilent Technologies) and an Agilent 2100 bioanalyzer. Genomic library preparation was carried out using the TruSeq DNA Sample Prep Kit v2 (Illumina, San Diego, CA, USA) according to manufacturer’s instructions. Genomic libraries were sequenced using the TruSeq Sequencing by Synthesis Kit v3-HS and the TruSeq Paired-End Cluster Kit v3-cBot-HS (Illumina) using three lanes of an Illumina HiSeq. 1000 sequencer according to the manufacturer’s instructions to generate 100-bp paired-end reads.

The sequencing reads were filtered using the following protocol: (i) Reads having more than 10% of undetermined bases or more than 50 bases with a phred score quality <7 were discarded using a custom script; (ii) For genomic libraries only, polymerase chain reaction (PCR) duplicates were removed with a custom script; (iii) Sequencing adapters were clipped using Scythe software ver. 0.980 (https://github.com/vsbuffalo/scythe); (iv) 3’ ends of reads were trimmed with a quality threshold of 20 spanning a window of 10 bases using Sickle ver. 0.940 (https://github.com/najoshi/sickle); and (v) Reads shorter than 20 bp were discarded. The heterozygosity rate was estimated for the CVT 71 clone using the GCE tool (https://arxiv.org/abs/1308.2012).

### Transcriptome assembly, annotation and transcript validations

An RNA library for annotation purposes was produced that pooled the RNA extracted from 27 samples (Table S3) using the TruSeq Stranded mRNA Library Prep Kit (Illumina) and processed with Duplex-Specific Thermostable Nuclease (DSN) in order to normalise the most abundant transcripts. Filtered RNA-Seq reads were assembled using Trinity ver. r2013–02–25^55^ with the default parameters and specifying the ‘–SS_lib_type RF’ parameter for the directionality of the library. The assembled transcript dataset was filtered of vectors using Seqclean (https://sourceforge.net/projects/seqclean/)^56^ and of redundant sequences and non-protein-coding assemblies using EvidentialGene ver. 2013.09.13 (http://arthropods.eugenes.org/EvidentialGene/). The filtered transcripts were incorporated into the PN40024 gene annotation using PASA ver. r2014-04-17^57^. Assembled transcripts not incorporated into the annotation were queried against the RefSeq ver. 2016–08 protein database using BLAST ver. 2.2.28^58^ and filtered of putative contaminants using MEGA ver. 4^59^, selecting only sequences assigned to the Viridiplantae clade. Comparison with other transcripts were performed by local alignment using BLAST ver. 36 × 1^60^. Putative novel gene models and selected non-aligning transcripts were functionally annotated by Blast2GO^61^.

For transcript validation, the RNA extracted from different ‘Nebbiolo’ clones and cultivars (Table S3 and S6) were DNase treated, converted in cDNA and amplified by end point RT-PCR following the protocol previously described^62^. Primer pairs were designed using Primer3 web ver. 4.0.0 software (http://primer3.ut.ee/) and reported in Table S13. Amplification products were purified using the Wizard^®^ SV gel and PCR Clean-Up System (Promega, WI, USA), and DNA was quantified using a NanoDrop 2000 spectrophotometer (Thermo Scientific). Sequencing was performed using the Big-Dye Terminator v1.1 Cycle Sequencing Kit (Applied Biosystems) following the manufacturer’s instructions. PCR products were purified using the DyeEx 2.0 Spin Kit (Qiagen, Germany) and analysed using a 3130 Genetic Analyzer capillary sequencer (Applied Biosystems).

### Genomic read alignment and SNV identification

Filtered reads were aligned to the *V*. *vinifera* PN40024 12x genome assembly as references using BWA ver. 0.6.2-r126^63^ with the default parameters. SNVs and short INDELs for each clone were detected using the GATK ver. 2.4.7. pipeline^64^: (i) Reads that aligned uniquely to the genome were selected by setting a mapping quality ≥ 1; (ii) Read duplicates were marked and read groups replaced using Picard tools ver. 1.96 (https://broadinstitute.github.io/picard/); (iii) Local reads were realigned with the GATK ‘IndelRealigner’ to minimise artefacts due to INDELs; and (iv) Variant calling was performed with GATK in ‘UnifiedGenotyper’ mode.

The biological effects of identified variants were predicted using SNPeff ver. 3.0^65^ on the V1 version of the annotation of the PN40024 genes (http://genomes.cribi.unipd.it/DATA/). Genes associated with SNVs were grouped using Blast2GO^54^. GO enrichment analysis was applied to gene variants in ‘Nebbiolo’ using the BiNGO 3.0 plug-in tool in Cytoscape ver. 3.2 as described by Maere *et al*.^26^. Over-represented PlantGOslim categories were identified using a hypergeometric test with a significance threshold of 0.05.

### SNV selection and validation

To filter the identified SNVs, those located in repetitive regions were excluded using both Bedtools^66^ and the database of *V*. *vinifera* V1-R repeats retrieved from the website of the CRIBI Biotechnology Center (http://genomes.cribi.unipd.it/). Spurious alleles were also discarded from the SNV set. To remove non-informative variants, we initially tried to exclude the low-confidence SNVs with the ‘Variant Filtration Tool’ of GATK using quality parameters suggested in ref.^46^: (i) phred-scaled quality score (QUAL) < 100; (ii) coverage <0.5 times the average coverage; iii) coverage >3 times the average coverage; (iv) strand bias (SB) > 0; (v) Fisher strand (FS) < 1; (vi) distance from the end of the read for reads with the mutated allele (ReadPosRankSumTest) <2 and >2.5; and (vii) minimum allele frequency (AF) < 0.2. The average coverage of filtered sequencing reads was estimated to be approximately 30- fold for each clone using Bedtools ver. 2.17.0; therefore, the maximum allowable coverage was initially set to 90 fold, and the minimum allowed was set to 15 fold. Since we noticed that some of these criteria were too stringent and other too permissive to allow a reliable identification of ‘Nebbiolo’ clones’ SNVs, we decided to use quality parameters of the set of validated SNVs to recalibrate the whole set of called variants. We thus discarded low-confidence SNVs according to the following parameters: (i) coverage <15; (ii) Fisher strand (FS) > 10; (iii) QualbyDepth <10; (iv) ReadPosRankSum >2. Moreover, based on characteristics of validated SNVs, we discarded homozygous alternative (or reference) SNVs which resulted with more than 2 reads mapped on the reference (or alternative) allele, to avoid retaining false positive variant calls.

Candidate SNVs for validation should also fulfil the following criteria: (i) the feasibility of designing TaqMan^®^ SNV Genotyping Assay probes (Applied Biosystems) within a 300–500-bp range including the polymorphism using the software Primer Express ver. 3.0 (Thermo Scientific, DE, USA); (ii) the manual review of the BAM alignment files for the exclusion of any possible artefacts (coverage verification and the visual inspection of reads for the actual presence of the polymorphism); and (iii) the alignment of the 300-bp region surrounding the SNV on the *V*. *vinifera* reference genome using BLASTN ver. 2.2.28+^58^, excluding sequences with multiple hits throughout the genome.

SNV validation was performed by the amplification of 300–500-bp genomic regions including the SNVs, followed by Sanger sequencing. Sequences flanking the SNV mutation were selected from the reference genome PN20024 and used to design primer pairs using Primer3 web ver. 4.0.0 software (http://primer3.ut.ee/). Primers were designed with the following criteria: (i) melting temperature between 52 and 65 °C; (ii) product size of 300–500 bp, (iii) primer length of 18–20 bp, (iv) and GC content between 30 and 70%. PCR amplifications were performed in a volume of 40 µL containing 80 ng of DNA, 1x KAPA Taq Buffer A containing 1.5 mM MgCl_2_ (KAPA Biosystems, MS, USA), 0.2 mM dNTPs, each primer at 0.5 µM and 0.5 U of KAPA Taq DNA polymerase (KAPA Biosystems). The PCR conditions included an initial denaturation step at 95 °C for 3 min followed by 35 cycles of denaturation (95 °C for 30 s), annealing (optimal temperature for each primer pair for 30 s), and extension (72 °C for 1 min). The final elongation step was carried out at 72 °C for 5 min. Amplification products were purified using the Wizard^®^ SV gel and PCR Clean-Up System (Promega, WI, USA), and sequenced as above described.

### SNV genotyping and data analysis

A total of 98 ‘Nebbiolo’ samples were analysed at 10 SNV marker locations (Table S11) using a Custom TaqMan^®^ SNV genotyping assay (Applied Biosystems). Among these samples, 40 accessions were clones officially registered in the Italian National Register of Grape Varieties and were sampled from the primary source plants. Fifty-eight accessions were sampled from vineyards in the Piedmont, Lombardy, and Aosta Valley regions. DNA was extracted from young leaves using a plant/fungi DNA isolation kit (Norgen Biotech Corp., ON, Canada). Chimerisms were investigated by extracting DNA from berry skin (L1 + L2) and berry flesh (L2) of the three sequenced clones. To avoid contamination, berry flesh was isolated by dissecting the cells between the berry skin and the tissue surrounding seeds as previously reported^19^. All samples were initially genotyped at six SSR markers^27^ in order to confirm the varietal identity of the samples. PCR amplifications and SSR analysis were performed according to the procedures described by Ruffa *et al*.^67^.

SNVs were analysed by qRT-PCR using specific TaqMan probes designed with Primer Express ver. 3.0 (Thermo Scientific, DE, USA) (Table S13). The amplification reaction was performed in a final volume of 10 μL containing 45 ng of DNA, following the manufacturer’s instructions. Allelic discrimination plots were constructed using the StepOne Plus system (Applied Biosystems) and CFX96 Detection System (Biorad), and the following amplification profile was used: 50 °C for 2 min; an initial denaturation cycle of 95 °C for 10 min; 45 cycles of 92 °C for 15 s and 60 °C for 1 min; and a final step of 60 °C for 30 s.

The genetic relationships among the different genotypes were investigated using two types of analysis. A UPGMA was used to construct and draw a dendrogram from the genetic similarity matrix using MEGA ver. 5.05^68^. Genetic distances (1,000 bootstraps) were computed as D = [1 − (proportion of shared alleles)] using Microsat software^69^. A median network was also constructed using the program Network ver. 4.5^70^.

### Data access

Raw sequenced reads can be found in the NCBI Sequence Read Archive under accession numbers SRR5626055, SRR5626056, SRR5626393 and SRR5626750. Variation data are downloadable as VCF files from http://ddlab.sci.univr.it/files/Nebbiolo/Nebbiolo.clones.filtered.SNVs.vcf. Nebbiolo CVT71 specific transcripts sequences (Table S5) together with PN40024 and novel genes annotations can be downloaded from, respectively, http://ddlab.sci.univr.it/files/Nebbiolo/Nebbiolo_CVT71_specific_transcripts.fasta and http://ddlab.sci.univr.it/files/Nebbiolo/Nebbiolo_CVT71_annotation.gff3.

## Electronic supplementary material

Supplementary information

Table S5

Table S7

Table S8

Table S9

Table S10

Table S11

Table S12

Table S13
