# Supplementary material for: Whole-genome sequencing and SNV genotyping of ‘Nebbiolo’ (Vitis vinifera L.) clones
Source: Sci Rep. 2017 Dec 11;7:17294. doi: 10.1038/s41598-017-17405-y (PMC5725591; doi:10.1038/s41598-017-17405-y)
Supplement: Supplementary file 1 — Supplementary information [file 41598_2017_17405_MOESM1_ESM.pdf]

Whole-genome sequencing and SNV genotyping of 'Nebbiolo' (*Vitis vinifera* L.) clones.

Giorgio Gambino[1a]*, Alessandra Dal Molin[2a], Paolo Boccacci[1], Andrea Minio[2], Walter Chitarra[1], Carla Giuseppina Avanzato[2], Paola Tononi[2], Irene Perrone[1], Stefano Raimondi[3], Anna Schneider[3], Mario Pezzotti[2], Franco Mannini[3], Ivana Gribaudo[3], Massimo Delledonne[2].

Institute for Sustainable Plant Protection, National Research Council (IPSP-CNR), Torino[1] and Grugliasco (TO)[3], Italy;

[2]Department of Biotechnology, University of Verona, Verona, Italy.

[a] These authors equally contributed to this paper.

Supplementary Figures

Figure S1. Putative ‘Nebbiolo’-specific transcripts expression analysis in a pool of tissues (Table S3) collected from ‘Nebbiolo’ CVT 71 in 2013 (N_2013), and in 2017 (Table S6) from ‘Nebbiolo’ CVT 185 and CVT 423 (N_2017), ‘Barbera’ (B) and PN40024 (P); -, water. The gene VIT_04s0044g00150 not specific for ‘Nebbiolo’ was used as control in order to verify the amplification in ‘Barbera’ (B) and PN40024 (P). Primers sequences are shown in Table S13.

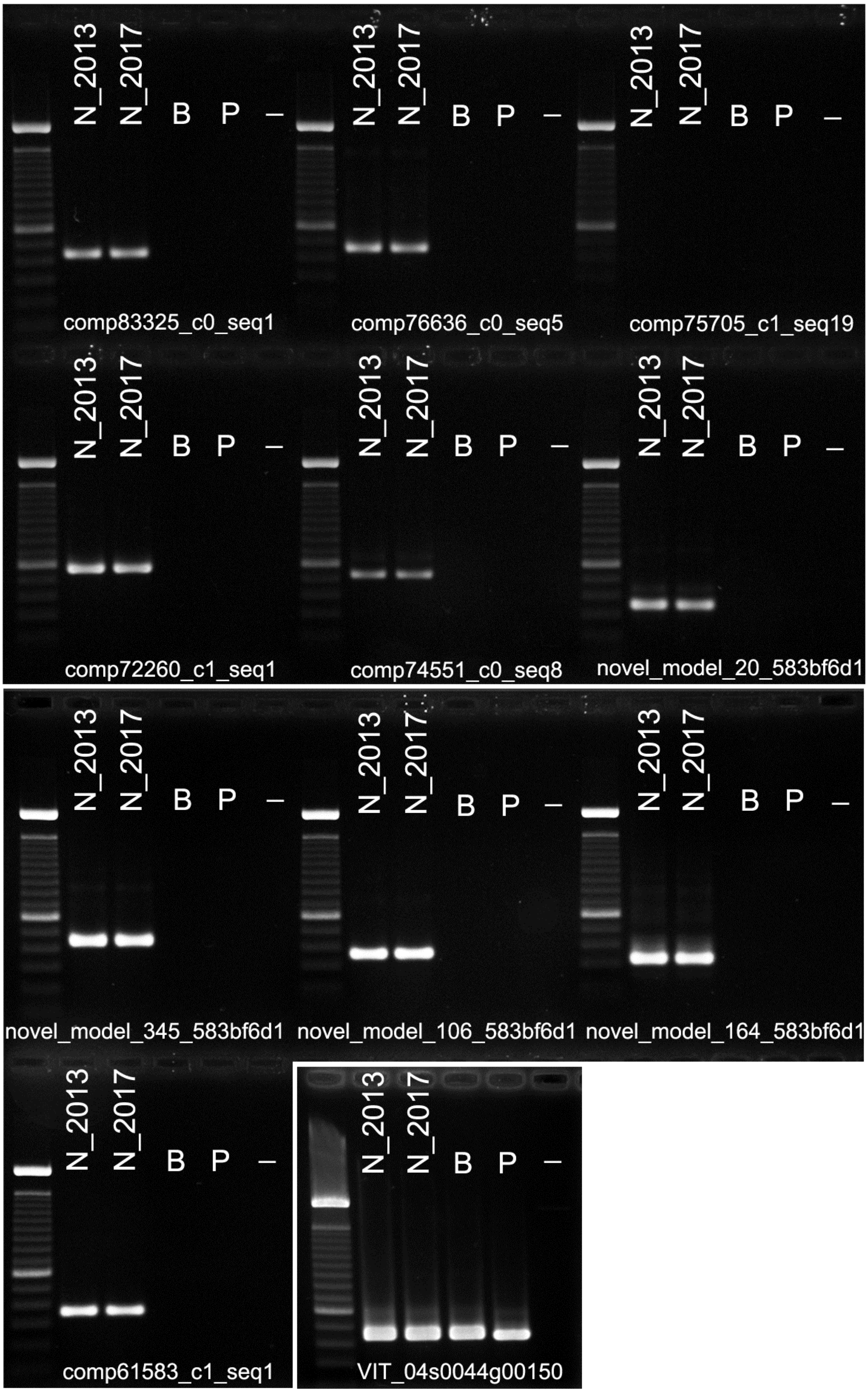

Figure S2. BinGO results for over-represented GO biological processes of all genes associated with SNVs shared by the three 'Nebbiolo' clones (FDR < 0.05). Coloured circles represent over-represented processes.

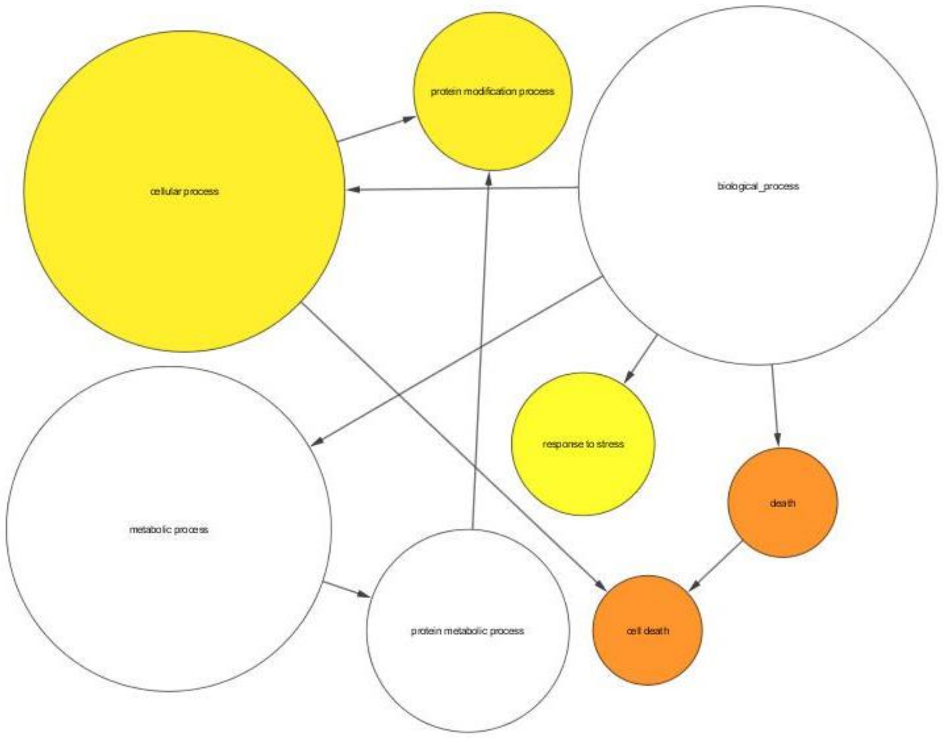

Figure S3. Diagrams showing the numbers of filtered putative clone-specific SNVs together with the numbers for protein-coding and high-impact SNVs obtained with SNPeff analysis.

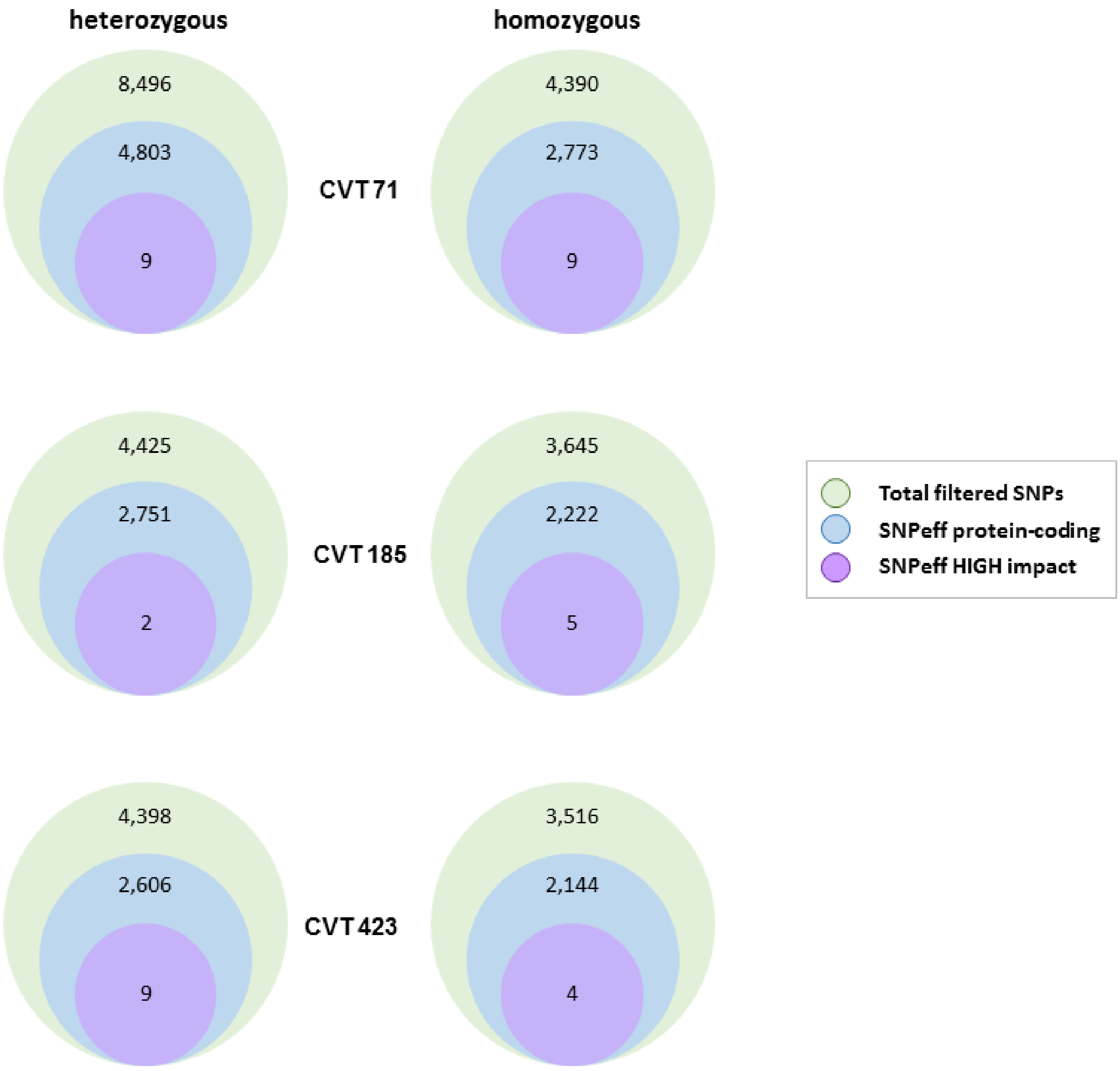

Figure S4. Output of TaqMan® SNV genotyping assays for Ne_SNV31, Ne_SNV33 and Ne_SNV62. For the three sequenced clones CVT 423, CVT 71 and CVT 185 were analysed berry skin (BS), berry flesh (BF) and leaf (L) in order to identify periclinal chimeras.

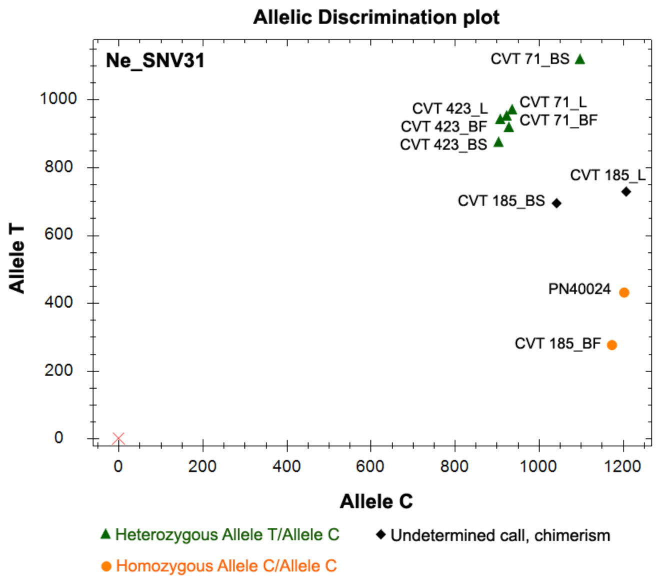

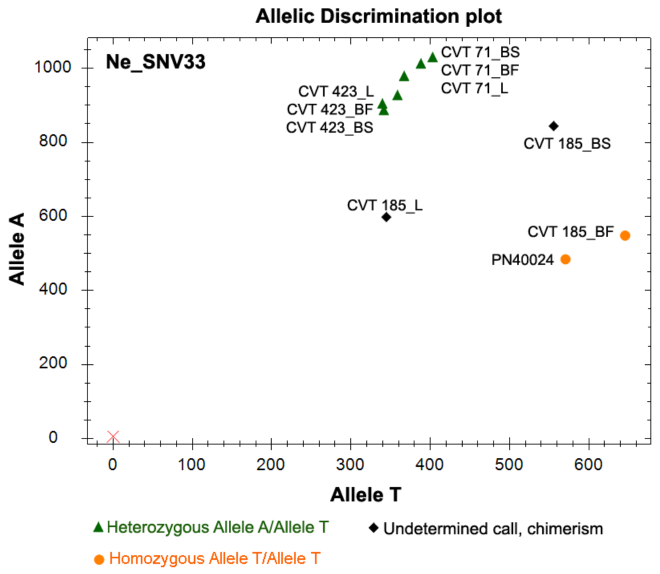

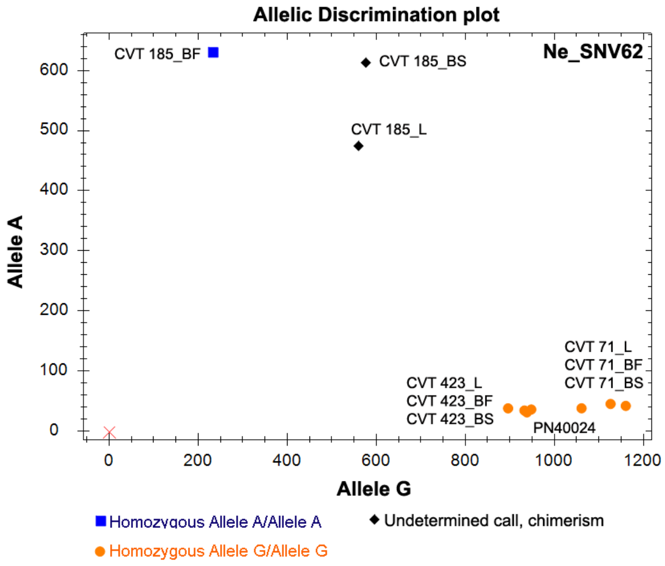

Supplementary Tables

Table S1. Description of the three 'Nebbiolo' clones used for the genome sequencing.

Characteristics	***CVT 71***	***CVT 185***	***CVT 423***
Origins	The mother plant was found in La Morra (CN, Italy) and belonged to the biotype "Michet". The clonal line was certified free from viruses according to the E.U. current regulations. The clone was registered in the Italian National Catalogue in 2001.	The mother plant was found in Barbaresco (CN, Italy) and belonged to the biotype "Lampia". The clonal line was certified free from viruses according to the E.U. current regulations. The clone was registered in the Italian National Catalogue in 2003.	The mother plant was found in Donnas (AO, Italy) and belonged to the biotype "Picoutener". The clonal line was certified free from viruses according to the E.U. current regulations. The clone was registered in the Italian National Catalogue in 2001.
Morphology	Medium-small leaves, five lobes, small and sharp teeth; good fertility, medium-large bunches (250 g on average) of short and cylindrical shape, none or short wings.	Small leaves, deeply five-lobed, medium sharped teeth; medium-low fertility, medium bunches (230 g on average) of medium-long pyramidal shape with a lateral wing.	Medium size leaves, deeply three or five-lobed, sharp teeth; medium fertility, small bunches (190 g on average) of medium-long cylindrical shape with short lateral wings.
Field attitudes	Medium-high vegetative vigour, medium-high yield (9 t/ha on average) and good environmental stability	Medium vegetative vigour, medium yield (7 t/ha on average) and medium environmental stability.	Low vegetative vigour, medium-low yield (6 t/ha on average) and medium-low environmental stability.
Wine characteristics	High degree of alcohol, balanced acidity, round body, intense ruby color with bright violet nuances due to the relatively rich dotation in three-substituted anthocyanins.	High degree of alcohol, high acidity, round body, ruby color with bright violet nuances due to the relatively rich dotation in three-substituted anthocyanins.	High degree of alcohol, balanced acidity, strong body, ruby color with slight violet nuances due to the lower dotation in three-substituted anthocyanins.

Table S2. Summary statistics of the sequencing of three ‘Nebbiolo’ clones.

Nebbiolo Clone	*# total fragments (100x2)*	*# filtered fragments (100x2)*	*# mapping fragments (100x2)*	*# uniquely mapping fragments (100x2)*
CVT 71	169,672,587	150,191,499	146,660,699 [97.65%]	141,488,796
CVT 185	99,911,238	91,387,604	85,197,798 [93.23%]	73,706,247
CVT 423	158,017,869	149,237,149	140,434,935 [94.10%]	135,311,040

Table S3. Plant material collected in 2013 from clone CVT 71 for *de novo* transcriptome characterization of ‘Nebbiolo’ by RNA-seq analysis.

Plant Material	***Phenological phases according to the E-L System modified by Coombe (1995)***
Winter wood	
Winter dormant buds	
Bud scales opening	E-L3
Bud after bud burst	E-L5, E-L7, E-L9
Mature leaves	E-L17, E-L27
Senescent leaves	E-L38
Roots (from potted plants)	
Green canes	E-L31
Flowers	E-L17, E-L23
Tendrils	E-L17, E-L27
Rachises	E-L23, E-L27, E-L31, E-L35, E-L38
Berries fruit set	E-L27
Berries pea size	E-L31
Berries veraison	E-L35
Berries harvest	E-L38
Seeds	E-L31, E-L35, E-L38

Table S4. Summary statistics of RNA-sequencing data of CVT 71 sample obtained by pooling of 27 different tissues.

Library Type	*Stranded + DSN normalized*
Sequencing Method	PE 2×101 bp
# fragments (100×2)	208,880,817
# filtered fragments (100×2)	123,629,502

Table S6. Plant material collected in 2017 from 'Nebbiolo' clones CVT 185 and CVT 423, PN40024 and 'Barbera' to validate the putative 'Nebbiolo'-specific transcripts identified from the *de novo* transcriptome assembly.

Plant Material	*Phenological phases according to the E-L System modified by Coombe (1995)*
Young leaves	E-L27
Mature leaves	E-L33
Senescent leaves	E-L37
Roots (from potted plants)	
Green canes	E-L33
Tendrils	E-L33
*Rachises**	E-L33, E-L35
*Berries bunch closure**	E-L33
*Berries veraison**	E-L35
*Seeds**	E-L33, E-L35

*organs not collected from PN40024.
